# Acute Posterior Multifocal Placoid Pigment Epitheliopathy With Associated Papillitis

**DOI:** 10.7759/cureus.35499

**Published:** 2023-02-26

**Authors:** Tatyana R Beketova, Kiersten Snyder, Alicia Jiang, Robert G. Josephberg

**Affiliations:** 1 Ophthalmology, New York Medical College, Valhalla, USA

**Keywords:** disc hemorrhage, covid-19 vaccination, macular edema, papillitis, corticosteroids, acute posterior multifocal placoid pigment epitheliopathy

## Abstract

The purpose of this study was to report the rare presentation of bilateral acute posterior multifocal placoid pigment epitheliopathy (APMPPE) and unilateral papillitis treated successfully with corticosteroid therapy. The methods used in this study include fundus photography and fluorescein angiography.

A 40-year-old female presented to the emergency room with decreased vision, headache, and photophobia with fundus examination findings of bilateral creamy placoid lesions in the posterior pole and unilateral papillitis, macular edema, and disc hemorrhages. Fluorescein angiography demonstrated early hypofluorescence corresponding to the placoid lesions followed by late, irregular hyperfluorescent staining. Optical coherence tomography revealed peripapillary and macular edema of the left eye. The patient was treated with two retrobulbar corticosteroid injections and a course of oral prednisone with improvement in fundus findings and visual acuity at follow-up examination six weeks from the presentation. The presence of optic nerve and macular edema in APMPPE suggests severe chorioretinal inflammation for which systemic and local corticosteroids are a reasonable treatment option.

## Introduction

Acute posterior multifocal placoid pigment epitheliopathy (APMPPE) is a self-limited inflammatory uveitis characterized by cream-colored placoid lesions localized at the retinal pigment epithelium (RPE), which causes acute central vision loss [1]. APMPPE affects both genders equally and usually occurs between the second and fourth decades of life [1]. It is typically bilateral, although the fellow eye may be involved days to weeks later after involvement of the initial eye [1]. This disorder was thought to primarily affect the RPE, however, with newer imaging modalities, investigators later suggested that inflammation of the choroidal vasculature was the primary driver [1,2]. In addition to the characteristic placoid lesions at the RPE, atypical features including serous retinal detachments, retinal vasculitis, and papillitis have been described in a handful of anecdotal reports [1,2].

Though the pathogenesis of APMPPE is still under debate, an inflammatory or autoimmune process is thought to play a role. APMPPE often follows a flu-like illness and has been associated with a variety of systemic vasculitic, infectious, and inflammatory processes [1,3]. It can also rarely occur after vaccinations for hepatitis B, meningococcus C, varicella, influenza, and coronavirus disease 2019 (COVID-19) [4-9].

There have been few reports in the literature that describe atypical features of APMPEE and fewer that report APMPPE presenting after COVID-19 vaccination [6]. Here, we present a case of APMPEE with atypical findings of papillitis with macular edema necessitating corticosteroid therapy, occurring two weeks after administration of the Pfizer-BioNTech COVID-19 booster vaccine.

## Case presentation

**Figure 1:**
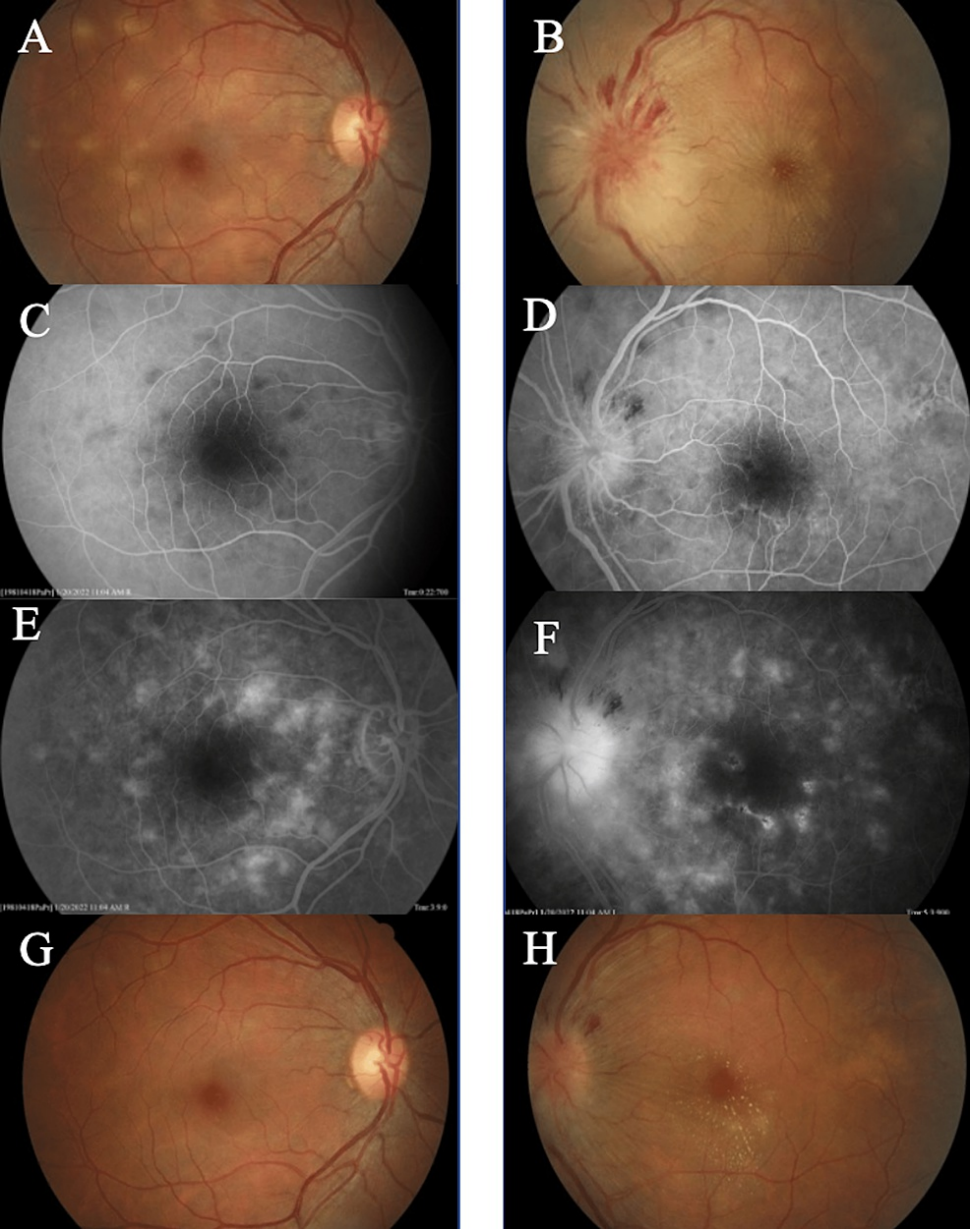
Fundus photography of right and left eyes. Right eye - (A) color fundus photo at presentation, multiple non-coalescing creamy-colored placoid lesions in the posterior pole. (C) Arteriovenous phase of fluorescein angiogram, multiple hypofluorescent lesions at the posterior pole. (E) Late phase of fluorescein angiogram, multiple hyperfluorescent lesions at the posterior pole. (G) Color fundus photo three weeks after presentation, placoid lesions resolved. Left eye - (B) color fundus photo at presentation, multiple non-coalescing creamy-colored placoid lesions in the posterior pole, severe papillitis with superior disc hemorrhages, and surrounding retinal edema. (D) Arteriovenous phase of fluorescein angiogram, multiple hypofluorescent lesions at the posterior pole. (F) Late phase of fluorescein angiogram, multiple hyperfluorescent lesions at the posterior pole, papillary leakage. (H) Color fundus photo three weeks after presentation, macular exudates, resolving disc hemorrhages, resolved placoid lesions.

A 40-year-old female presented to the emergency department with a three-day history of decreased vision of the left eye with associated photophobia and headaches. Past medical history was significant for type 2 diabetes mellitus, hyperlipidemia, and hypothyroidism. The patient had obtained the Pfizer-BioNTech COVID-19 booster vaccine 14 days prior to presentation. Best-corrected visual acuity (BCVA) was 20/20 in the right eye and count fingers at 6 feet in the left eye. Intraocular pressures were within normal limits. Pupils were round and reactive, without afferent pupillary defect bilaterally. Confrontational visual fields were full in the right eye and showed temporal visual field cut of the left eye. Slit-lamp examination of the right eye revealed trace cell without flare in the anterior chamber, 2+ nuclear sclerotic cataracts, and 1+ vitreous cells. Slit lamp examination of the left eye revealed trace cell with flare in anterior chamber, 2+ nuclear sclerotic cataracts, and clear vitreous. On dilated fundus examination, multiple non-coalescing creamy-colored placoid lesions were noted throughout the posterior pole of both eyes (Figures 1A, 1B). Examination of the left optic nerve showed severe papillitis with superior disc hemorrhages and surrounding retinal edema (Figure 1B). Right optic nerve was unremarkable (Figure 1A). Optical coherence tomography (OCT) of the right nerve and macula was flat. OCT of the left macula revealed significant retinal thickening and edema of the outer retinal layers, greater nasally than temporally. OCT of the left nerve revealed extensive thickening of the retina nerve fiber layer. Magnetic resonance imaging (MRI) brain and orbits was significant for contrast enhancement within the posterior aspect of the left globe at the optic nerve sheath insertion. Laboratory workup including cerebrospinal fluid (CSF) culture and cytology, blood culture, complete blood count (CBC), complete metabolic panel (CMP), erythrocyte sedimentation rate (ESR), Bartonella polymerase chain reaction (PCR), immunoglobulin G (IgG) and immunoglobulin M (IgM), Venereal Disease Research Laboratory (VDRL), rapid plasma reagin (RPR), QuantiFERON-TB Gold Plus (Hilden, Germany: QIAGEN); viral panel including herpes simplex virus (HSC), cytomegalovirus (CMV), and varicella zoster virus (VZV) was non-contributory. On follow-up examination one week later, fluorescein angiography (FA) revealed lesions with early hypofluorescence corresponding to the placoid lesions followed by late, irregular hyperfluorescent staining (Figures 1C-1F). The left eye was treated with a retrobulbar injection of triamcinolone 20 mg (0.5 mL of 40 mg/mL suspension), and prednisolone acetate 1% drops were started four times a day in both eyes. On re-examination two weeks from presentation, the left eye visual acuity had improved to 20/70 and was treated with a retrobulbar injection of dexamethasone 6 mg (1.5 mL of 4 mg/mL) in the left eye. After confirming that the patient's type 2 diabetes was adequately controlled, the patient was also started on an oral prednisone taper of 10 mg twice daily for one week, then 10 mg daily for two weeks. On examination three weeks after the presentation visual acuity had improved to 20/40 in the left eye with slit lamp examination revealing a quiet anterior chamber and vitreous (Figures 1G, 1H). The patient reported that the headaches had resolved.

## Discussion

Acute posterior multifocal placoid pigment epitheliopathy (APMPPE) is an inflammatory disease involving the retina and choroid [2]. Choroidal inflammation and vasculitis from a delayed-type hypersensitivity reaction are thought to be the primary inciting agent in the disease, with retinal pigment epithelium damage occurring secondarily [2,10]. APMPPE is characterized by cream-colored placoid lesions which demonstrate early hypofluorescence and late hyperfluorescence on fluorescein angiography (FA) [1]. Although our patient had these characteristic findings, her fundus examination also showed unilateral macular edema and papillitis with disc hemorrhages and vasculitis. This was initially a diagnostic dilemma and prompted a work-up to rule out infectious etiologies of neuroretinitis, which was negative. Papillitis in APMPPE is rare, with 14 cases published in the literature to date [1,2,10-12]. The presence of papillitis may indicate a more severe inflammation of the chorioretinal vasculature than is usually seen in APMPPE.

In this case, the severity of the left-sided papillitis coupled with the presence of significant macular edema prompted treatment with corticosteroids. Our patient’s visual acuity improved from counting fingers OS at initial presentation to 20/40 OS after a series of triamcinolone and dexamethasone injections into the left retrobulbar space after three weeks of follow-up. Local and systemic steroids have previously been successfully employed in cases of foveal-involving APMPPE in the active phase, and may even play a role in non-foveal APMPPE [9,13-15]. In a retrospective case series of 19 patients with APMPPE, Papasavvas et al. demonstrated superior outcomes in both visual acuity and visual fields in eyes treated with corticosteroids, regardless of foveal involvement [13]. Although the efficacy of corticosteroids on APMMPE-related visual impairment is yet to be tested in a clinical trial, close monitoring and prompt corticosteroid intervention should be standard of care in APMPPE due to the lack of predictability of the evolution of the disease [13].

APMPPE has been shown to occur as a post-viral syndrome and in association with systemic inflammatory or immune-mediated conditions (e.g., thyroiditis, erythema nodosum, granulomatosis with polyangiitis, polyarteritis nodosa, nephritis, sarcoidosis, ulcerative colitis, and central nervous system vasculitis) [1]. It has also been correlated with vaccination for hepatitis A and B, polio, tetanus, meningococcal, varicella, influenza, and COVID-19 [4,6-9,16]. In our patient, visual changes first occurred 11 days after the administration of the booster dose of the Pfizer-BioNTech COVID-19 vaccine. COVID-19 vaccination has been linked to systemic inflammatory conditions, such as myocarditis and pericarditis, and various other chorioretinal inflammatory conditions, such as multifocal choroiditis, posterior uveitis, acute macular retinopathy, and multiple evanescent white dot syndrome (MEWDS) [16-20]. It is important to note that although systemic and infectious causes were ruled out in our patient, we cannot definitively attribute the patient’s COVID-19 vaccine to her subsequent development of APMPPE.

## Conclusions

This case presents an atypical presentation of bilateral APMPPE with unilateral papillitis, macular edema, and disc hemorrhages, which is suggestive of a more severe chorioretinal vasculitis. The patient was treated successfully with topical, retrobulbar, and oral corticosteroids. Although it is not possible to predict whether our patient’s vision would have still recovered without intervention, we determined corticosteroid therapy to have a high benefit and low-risk profile given the level of optic nerve and macular involvement. This case highlights the importance of close monitoring and low threshold for corticosteroid treatment in cases of severe foveal-involving APMPPE.
